# A betacoronavirus multiplex microsphere immunoassay detects early SARS-CoV-2 seroconversion and antibody cross reactions

**DOI:** 10.21203/rs.3.rs-105768/v1

**Published:** 2020-11-24

**Authors:** Eric Laing, Spencer Sterling, Stephanie Richard, Nusrat Epsi, Shreshta Phogat, Emily Samuels, Lianying Yan, Nicole Moreno, Christian Coles, Matthew Drew, Jennifer Mehalko, Caroline English, Scott Merritt, Katrin Mende, Kevin Chung, G. Clifton, Vincent Munster, Emmie de Wit, David Tribble, Brian Agan, Dominic Esposito, Charlotte Lanteri, Edward Mitre, Timothy Burgess, Chritopher Broder

**Affiliations:** Uniformed Services University; Uniformed Services University of the Health Sciences; Uniformed Services University/Henry M. Jackson Foundation; Uniformed Services University/Henry M. Jackson Foundation; Uniformed Services University/Henry M. Jackson Foundation; Uniformed Services University/Henry M. Jackson Foundation; Uniformed Services University of the Health Sciences; Uniformed Services University/Henry M. Jackson Foundation; Uniformed Services University/Henry M. Jackson Foundation; Frederick National Laboratory for Cancer Research; Frederick National Laboratory for Cancer Research; Uniformed Services University/Henry M. Jackson Foundation; Uniformed Services University/Henry M. Jackson Foundation/Brooke Army Medical Center; Uniformed Services University/Henry M. Jackson Foundation/Brooke Army Medical Center; Uniformed Services University; Brooke Army Medical Center; National Institute of Allergy and Infectious Diseases; National Institute of Allergy and Infectious Diseases; Uniformed Services University; Uniformed Services University/Henry M. Jackson Foundation; Frederick National Laboratory for Cancer Research; Uniformed Services University; Uniformed Services University; Uniformed Services University; Uniformed Services University of the Health Sciences

**Keywords:** SARS-CoV-2, multiplex microsphere-based immunoassay, seroconversion

## Abstract

Sensitive and specific SARS-CoV-2 antibody assays remain critical for community and hospital-based SARS-CoV-2 surveillance. Here, we developed and applied a multiplex microsphere-based immunoassay (MMIA) for COVD-19 antibody studies that incorporates spike protein trimers of SARS-CoV-2, SARS-CoV-1, MERS-CoV, and the seasonal human betacoronaviruses, HCoV-HKU1 and HCoV-OC43, that enables measurement of off-target pre-existing cross-reactive antibodies. The MMIA performances characteristics are: 98% sensitive and 100% specific for human subject samples collected as early as 10 days from symptom onset. The MMIA permitted the simultaneous identification of SARS-CoV-2 seroconversion and the induction of SARS-CoV-2 IgG antibody cross reactions to SARS-CoV-1 and MERS-CoV. Further, synchronous increases of HCoV-OC43 IgG antibody levels was detected with SARS-CoV-2 seroconversion in a subset of subjects for whom early infection sera were available prior to their SARS-CoV-2 seroconversion, suggestive of an HCoV-OC43 memory response triggered by SARS-CoV-2 infection.

## Introduction

Severe acute respiratory syndrome coronavirus-2 (SARS-CoV-2) is a novel zoonotic positive-sense, single-stranded, RNA virus responsible for the third viral pandemic of the 21^st^ century, and the third zoonotic coronavirus outbreak in the past 20 years (1, 2). At this time, SARS-CoV-2 has globally caused 43 million COVID-19 cases and over 1 million COVID-19 related deaths. A major concern of the ongoing SARS-CoV-2 pandemic have been frequent reports of waning virus-specific antibody levels, with several studies reporting decay to undetectable levels within just a few months after infection (3–5). While this is a measurable feature of an antibody response, subsequent studies detected little decay of antibodies three to four months after infection (6, 7) and for as long as seven months (8); it is also possible that current assays lack the sensitivity required to detect lower levels of long-lived SARS-CoV-2 specific antibodies. To date, a variety of antibody tests have been developed with 38 tests granted Emergency Use Authorization (EUA) by the U.S. Food and Drug Administration. The majority of these tests assess for antibodies against the coronavirus spike (S) envelope glycoprotein, the primary target of virus-neutralizing antibodies (9), in either its native-like oligomer conformation, or against one of its protein subunits or domains. In general, most S glycoprotein antigen-based assays report the ability to detect antibodies in 65–70% of infected individuals 8 – 14 days after symptom onset, with positivity rates over 90% not occurring until 2 to 3 weeks after symptom onset (10).

In this study, we describe the development, characterization, and utility of a betacoronavirus (β-CoV) multiplex microsphere-based immunoassay (MMIA) for COVID-19 serology studies. To optimize sensitivity and specificity for measuring SARS-CoV-2 spike reactive antibodies, the MMIA included prefusion stabilized S glycoprotein ectodomain trimers of SARS-CoV-2, SARS-CoV-1, MERS-CoV, and the seasonal human coronaviruses (HCoV), HCoV-HKU1 and HCoV-OC43. The MMIA enabled the simultaneous measurement of relative antibody quantities against each of these medically-relevant betacoronaviruses. We hypothesized that this approach would result in a highly sensitive and specific assay for detecting SARS-CoV-2 specific antibodies through two mechanisms. Fifirst, the Luminex xMAP-based platform has a large dynamic range and has been shown to be more sensitive than ELISA for the detection of antibodies to other viral infections (11–13). Second, given the high seroprevalence of the common human β-CoVs (14–16), cross-reactive antibodies present in subject samples (17, 18) could be concurrently measured and accounted for in a multiplex approach. By testing for S glycoprotein reactive antibodies to SARS-CoV-2 in the presence of HKU1 and OC43 S glycoproteins, the MMIA assay controls for off-target pre-existing cross-reactive β-CoVs antibodies, thus enhancing specific SARS-CoV-2 antibody detection. Additionally, the simultaneous incubation of serum with S glycoproteins from all the relevant β-CoVs may enable a lower threshold for SARS-CoV-2-specific antibody positivity.

Utilizing serum samples from an experimentally challenged non-human primate (NHP) model, together with human sera from subjects confirmed to have SARS-CoV-2 infection and from subjects confirmed to have other coronavirus infections collected prior to 2018, we report the sensitivity and specificity performances for this assay strategy. Serum samples from rhesus macaques experimentally infected with SARS-CoV-2 demonstrated that SARS-CoV-2 S glycoprotein IgG seroconversion was detectable by 10 days post infection (dpi), consistent with other reports demonstrating anti-S glycoprotein IgG seroconversion between 3 and 14 dpi (19–22). We then evaluated serum samples from SARS-CoV-2 RTPCR positive subjects collected 10 days after symptom onset and report 98% sensitivity for SARS-CoV-2 S glycoprotein IgG antibody detection in humans at that time point. We also examined differences in SARS-CoV-2 antibody reactivity between widely used antigens: SARS-CoV-2 prefusion stabilized S glycoprotein ectodomain trimer and a monomeric receptor-binding domain (RBD). High seroprevalence of seasonal HCoV OC43 and HKU1, ranging from 97 – 98% and 55 – 89%, respectively, was observed across both pre-2019 sera and current SARS-CoV-2 negative subject serum samples. This MMIA strategy will also enhance investigations of the interplay of pre-existing seasonal HCoV antibodies on SARS-CoV-2 IgG duration, COVID-19 symptom presentation, and disease severity. Preliminary data using this multiplex serology strategy demonstrates that SARS-CoV-2 infection can stimulate an IgG antibody response that is cross-reactive with SARS-CoV-1 and MERS-CoV S glycoproteins, as well as an apparent anamnestic antibody response to OC43 S glycoprotein similar to back-boosting seen with influenza viruses (23).

## Results

### Comparison of MMIA and ELISA for SARS-CoV-2 IgG antibody detection

We fifirst established our ability to detect SARS-CoV-2 IgG and monitor SARS-CoV-2 seroconversion with sera collected from SARS-CoV-2 infected NHP. Purified IgG from SARS-CoV-2 infected NHP collected 21 dpi were pooled and spiked into NHP negative sera, and the MMIA was quantitatively characterized for IgG polyclonal reactivity revealing SARS-CoV-2 spike antibody MFI curve linearity between 0.625 – 5.0 μg/ml or 3690 – 20,354 MFI (Figure S1). To investigate the effects of the increased dynamic range facilitated by Luminex xMAP-based multiplexing systems on MMIA sensitivity, we compared end-point titers by both ELISA and MMIA. In an ELISA, SARS-CoV-2 positive NHP sera end-point titers ranged from 1,000 to 2,000 (Figure 1A), consistent with reported ELISA titers for these animals (19). In the MMIA, the ability to detect serially diluted IgG antibodies was 4- to 8-fold greater than ELISA with end-point titers ranging from 4,000 (n= 2) to 16,000 (n= 1) (Figure 1B).

### Seroconversion in a non-human primate model

Next, we monitored SARS-CoV-2 seroconversion with longitudinal NHP serum samples. SARS-CoV-2 spike protein reactive IgG antibody seroconversion was observed in all four NHP 10 dpi (Figure 2A). We also investigated SARS-CoV-2 IgM antibody seroconversion and detected IgM level above baseline in two NHP by 7 dpi; all four NHP had detectable IgM by 10 dpi (Figure 2B). Notably, IgG antibody from SARS-CoV-2 challenged NHP did not significantly react with spike proteins from other β-CoVs, SARS-CoV-1, MERS-CoV or HCoVs, included in the MMIA (Figure 2A), whereas, a varying degree of IgM cross-reactivity was observed (Figure 2B). Additionally, high baseline IgM reactivity to SARS-CoV-2 RBD at 0 dpi inhibited our ability to ascertain the dpi where seroconversion could be observed with this SARS-CoV-2 antigen (Figure 2B).

### Archival sera from subjects with PCR-confirmed seasonal coronaviruses exhibit cross-reactivity with SARS-CoV-2 spike protein

Despite low sequence similarity and identity between SARS-CoV-2 spike protein and seasonal HCoV spike proteins, antibody cross-reactivity with SARS-CoV-2 proteins has been observed (17, 18). To determine whether prior infection with seasonal HCoV induces antibodies that cross-react with SARS-CoV-2, we assayed sera from collected prior to 2019 from human subjects with PCR-confirmed seasonal HCoVs: the Acute Respiratory Infection Consortium Natural History Study (ARIC) (24). When setting a cut-off for positivity at three times the mean MFI obtained for a mock antigen preparation-coupled microsphere, we observed that 8.89% (4/45) of archived serum samples from HCoV PCR-positive subjects cross-reacted with SARS-CoV-2 spike protein (Figure 3A-D). Cross-reactivity between HCoV-induced antibodies with SARS-CoV-2 spike protein was observed in subjects that were PCR-positive for OC43 (1/16), HKU1 (1/6) and 229E (2/10) infection.

### Multiplex microsphere-based immunoassay performance

To control for pre-existing HKU1 and OC43 spike protein reactive antibody cross-reactivity with SARS-CoV-2 spike protein, rather than using a cutoff of three times the mean MFI obtained for mock antigen, IgG and IgM antibody threshold cutoffs were established with HCoV PCR-positive convalescent sera from the ARIC human sera cohort (Figure S2A–B). Conventional 99.7% probabilities, mean and three standard deviations higher, threshold cutoff values for MFI were first employed to distinguish SARS-CoV-2 positive and negative IgG/IgM antibodies. Next, serum samples (n= 422) from subjects enrolled in the ongoing Epidemiology, Immunology, and Clinical Characteristics of Emerging Infectious Diseases with Pandemic Potential (EPICC) protocol were screened for SARS-CoV-2 IgG and IgM antibody reactivity. A ROC curve analysis with SARS-CoV-2/EPICC serum samples was used to identify the MFI value associated with 100% specificity for SARS-CoV-2 reactive IgG and IgM. The ROC analysis EPICC serum samples determined IgG antibody threshold cutoff with 100% specificity, 4853 MFI (Figure S3), was nearly identical to the 99.7% probability threshold cutoff set with the pre-2019 ARIC cohort, 4910 MFI.

Pre-2019 ARIC human serum samples (n= 84), representing HCoV PCR-positive acute sera, rhinovirus PCR-positive acute/convalescent sera, and acute/convalescent sera from ‘no pathogen detected’ subjects, did not react with the SARS-CoV-2 spike protein above either the established 99.7% probability threshold cutoff or ROC threshold cutoff for IgG antibody (Figure 4A). A similar approach was taken to establish threshold cutoffs for IgM antibody (Figure 4B). A receiver operating characteristic (ROC) curve analysis established a threshold, 1445 MFI (Figure S4), which was more conservative than the 99.7% probability threshold cutoff, 840 MFI (Figure S2B), and was chosen to identify SARS-CoV-2 positive IgM. Subjects that were PCR confirmed as SARS-CoV-2 negative with serum samples collected < 30 days post-symptom onset (dpso) displayed no IgG or IgM reactivity with SARS-CoV-2 spike protein (Figure 5A).

Utilizing serum samples from both EPICC enrollees and enrollees from a another protocol (COVID-19 Antibody Prevalence in Military Personnel Deployed to New York (CAMP-NYC)), we assessed the sensitivity of the MMIA to detect IgG and IgM seroconversion. Sera from subjects with SARS-CoV-2 infection were screened for IgG and IgM antibody reactivity with β-CoV spike proteins in our MMIA. As SARS-CoV-2 seroconversion was detected by 10 dpi in NHPs, MMIA SARS-CoV-2 spike protein IgG sensitivity for human serology was evaluated in SARS-CoV-2 PCR-confirmed subjects ≥ 10 dpso. SARSCoV-2 reactive IgM and IgG antibodies were only detected above our threshold cutoffs in the PCR positive subjects enrolled at military hospitals or the Javits Center field hospital; cross-reactive antibodies to SARS-CoV-1 and MERS-CoV were observed in SARS-CoV-2 PCR positive and IgG positive subjects, but not SARS-CoV-2 PCR negative subjects (Figure 5A-B).

Performance assessments of this β-CoV MMIA for SARS-CoV-2 spike protein IgG and IgM detection are included in Table 1. SARS-CoV-2 spike protein reactive IgG antibody detection was calculated with 95% confidence intervals (CI) as follows, sensitivity = 98.06% (94.45% – 99.60% CI), specificity= 100% (96.82% – 100.00% CI). Assuming a US disease prevalence of 1.0%, the positive predictive value (PPV) = 100.00%, and negative predictive value (NPV) = 99.98% (99.94% - 99.99% CI). SARS-CoV-2 spike protein reactive IgM detection sensitivity was lower than IgG, with performance analysis conducted with serum samples collected ≥ 7 days post-symptom onset, sensitivity= 78.10% (68.97% - 85.58% CI), specificity 100.00% (96.95 – 100.00%), PPV= 100.00. When comparing spike protein and RBD, notably, utility of SARS-CoV-2 RBD for IgG detection had a reduced sensitivity (87.10%, 80.78% - 91.94% CI) (Table S1).

To assess β-CoV MMIA precision, three positive and one negative samples were tested over five independent experiments, with at least two distinct in-house antigen-coupled bead lots and serum sample freeze-thaws. Coefficient of variations (CV) were calculated for all three positive samples and remained < 20% (Figure S5). Although the negative sample had a > 20%, the MFI never went above the threshold cutoff for positive IgG across five independent tests.

### COVID-19 subject antibody reactivity in a betacoronavirus MMIA

HKU1 and OC43 IgG antibodies were observed in pre-2019 sera (Figure 4A) and serum samples from SARS-CoV-2 PCR negative subjects (Figure 5A), with seroprevalence of 72% and 98%, respectively. We further investigated SARS-CoV-2 *de novo* IgG antibody cross-reactivity with SARS-CoV-1 and MERS-CoV spike proteins (Figure 5B), by combining serology data from pre-2019 ARIC sera and SARS-CoV-2 PCR negative subjects, and comparing to SARS-CoV-2 PCR positive subjects. IgG antibody levels detected with all five β-CoV spike proteins in the SARS-CoV-2 PCR positive patient serum samples were significantly higher than those in pre-2019 ARIC serum samples and SARS-CoV-2 negative patient serum samples (Figure 6A-E). These levels of SARS-CoV-1, MERS-CoV, HKU1 and OC43 reactive IgG antibodies after SARS-CoV-2 infection provide compelling evidence that SARS-CoV-2 stimulates a polyclonal antibody response containing cross-reactive antibodies that can bind to spike proteins from closely and distantly-related β-CoVs. Further, a MMIA analysis of available longitudinal serum samples provided a novel glimpse of potential differences in cross-reactive antibody responses. Maximum OC43 antibody levels (blue arrow) are reached at earlier dpso after SARS-CoV-2 infection than are SARS-CoV-1 and MERS-CoV-2 levels (red arrow), suggesting that SARS-CoV-2 stimulates an OC43 humoral memory response in some subjects, independent of the SARS-CoV-2 *de novo* IgG response that is cross-reactive with MERS-CoV and SARS-CoV-1 spike proteins (Figure 7A-D). Further, OC43 reactive IgG responses were observed to wane in parallel with SARS-CoV-2 IgG (Figure 7B-C), suggesting a temporal linkage between these β-CoV specific IgG responses.

## Discussion

In this study, we have demonstrated that use of a multiplex microsphere-based immunoassay (MMIA) built using Luminex xMAP-based technology in which individual microspheres are bound to pre-fusion stabilized S glycoprotein trimers of SARS-CoV-2, SARS-CoV-1, MERS-CoV, and each of the two seasonal β-CoVs enables highly sensitive and specific detection of SARS-CoV-2 IgG antibodies. In contrast to commercial ELISA and lateral flow assays for SARS-CoV-2 IgG, which typically have a sensitivity in the range of 65–70% up to 14 days after symptom onset (10), the MMIA has a sensitivity of 98% at just 10 days after symptom onset in PCR-confirmed cases of SARS-CoV-2 infection. Use of this highly sensitive MMIA will enhance studies on the kinetics of humoral responses to SARS-CoV-2 infection.

We hypothesize that the high sensitivity and specificity of this assay for SARS-CoV-2 serology is due both to the physics of the Luminex xMAP-based platform, which enables a high dynamic range of measurement, as well as its multiplexing design with CoV S glycoproteins. Multiplex microsphere-based immunoassays have been shown to be more sensitive than standard ELISA for SARS-CoV-2 antibody detection (25) and several other virus infections, including Lassa virus, Ebola virus, and simian immunodeficiency virus (11–13). Additionally, and perhaps more importantly, simultaneously incubating serum against spike proteins of seasonal HCoVs appears to enable the establishment of a lower threshold of positivity for detection of SARS-CoV-2 specific antibodies. Given the presence of cross-reactive antibodies, assays that only test for antibodies against the SARS-CoV-2 spike protein may have to utilize a high signal threshold as a cut-off for positivity to reduce false positive rates. By incubating serum against multiple CoV spike proteins, the MMIA platform would allow for the preferential binding of cross-reactive antibodies to the antigens of the CoV against which they were initially induced, thus enabling a lower, and apparently more sensitive, cut-off for the detection of SARS-CoV-2 specific antibodies.

Early in the COVID-19 pandemic, SARS-CoV-2 IgG seroconversion was surprisingly detected early after exposure and sometimes in parallel with IgM seroconversion (26–28). The temporal window to capture SARS-CoV-2 IgM is shorter than IgG, and with IgG seroconversion occurring 10 dpi in NHP SARS-CoV-2 disease models, and detectable as early as 7 dpso in subjects, there appears little benefit for continued SARS-CoV-2 IgM detection (Table S2). As we placed no upper limit on the dpso of the first serum collection included in performance analysis, the IgM sensitivity is lower than IgG, driven by outpatient enrollments in the EPICC, IDCRP-085 protocol with an average 28 dpso that were IgG positive, but IgM negative. Although less sensitive and less specific than IgG detection, the benefit of IgM detection may lay in its ability to place a temporal window on SARS-CoV-2 exposure in asymptomatic IgG positive individuals, which would be particularly useful for cross-sectional studies of seroprevalence.

Conservation of epitopes present in the prefusion stabilized native-like trimeric S glycoprotein oligomers are the likely major factor in the observed cross reactions between the CoV S glycoproteins. Since the RBD protein is only a domain within the S1 subunit of the S glycoprotein and lacks potentially conserved epitopes with seasonal HCoV S glycoproteins, its utility in antigen-based immunoassays confers specificity for SARS-CoV-2 and is thus employed in several antibody tests (29–32). We noted that in our β-CoV MMIA, the commercially sourced RBD protein, which shares an equivalent number of protein residues with the expressed RBD protein used in a microsphere-based immunoassay developed by the Ragon Institute of MGH, MIT and Harvard (25), had a higher threshold cutoff that limited IgG detection sensitivity when compared to the spike protein trimer. This reactivity to RBD may be driven by artificial epitopes, exposure of epitopes otherwise inaccessible within the context of the native-like S glycoprotein trimer or a product of microsphere coupling.

Immunoassay detection of IgG antibodies that can bind to RBD has been used as a surrogate for neutralization tests which require cell-culture, pseudoviruses, or biosafety-containment and wild-type SARS-CoV-2 (30, 33, 34). SARS-CoV-2 neutralizing antibodies target the S glycoprotein S1 subunit, particularly the RBD and N-terminal domains, and sterically interfere with human ACE-2 receptor interaction (35–39). However, SARS-CoV-2 and MERS-CoV neutralizing monoclonal antibodies have been identified that binds epitopes that do not interfere with receptor engagement (40, 41). Furthermore, nonneutralizing antibody-mediated protection has been observed in other virus infections, including HIV and Ebola virus (42, 43). Given the relative poor performance of this RBD protein in this β-CoV MMIA, and our ability to capture the full-breadth of the humoral response, e.g., RBD-binding, neutralizing and non-neutralizing, to SARS-CoV-2 infection with the native-like spike protein trimer (44), future studies will exclude the monomeric RBD antigen from the multiplex strategy.

Importantly, the MMIA approach provided additional antibody detection data on IgG cross-reactivity with SARS-CoV-1 and MERS-CoV, as well as an anamnestic antibody, or back-boosting, OC43 response in some subjects for whom OC43 antibody levels increased coincidentally with SARS-CoV-2 seroconversion. Conserved cross-neutralizing epitopes between SARS-CoV-1 and SARS-CoV-2 S glycoproteins have been identified (40, 45), whether SARS-CoV-2 induced *de novo* IgG antibody responses to SARS-CoV-1 and MERS-CoV spike proteins detected with this MMIA strategy are retained after affinity maturation, or are cross-neutralizing requires further investigation. As *de novo* IgG cross-reactivity with OC43 spike protein was not observed in SARS-CoV-2 challenged NHPs (Figure 2A), the presence of humoral memory to seasonal HCoV appears necessary to drive cross-reactivity. The observed increases in OC43 reactive IgG antibodies may be anamnestic stimulation or back-boosting of OC43 memory B cells through conserved epitopes in the SARS-CoV-2 S glycoprotein. Back-boosting stimulation of a cross-reactive memory response might also explain cases of synchronous SARS-CoV-2 IgM and IgG seroconversion and IgG seroconversion prior to IgM (46).

Overall, the OC43 S glycoprotein only shares 30 to 40% amino acid sequence identity/similarity with SARS-CoV-2 S glycoprotein (18). The S1 subunit, wherein resides the RBD, has more sequence variance among OC43 and SARS-CoV-2, in contrast to the S2 subunit heptad repeat regions where amino acid sequence similarity is between 50 to 75%. It would seem unlikely that a SARS-CoV-2 *de novo* IgG response would result in cross-reactive antibodies that would bind at immunoassay saturation to distantly-related seasonal β-CoVs. Further, the *de novo* generation of cross-reactive IgG to SARS-CoV-1 and MERS-CoV was not detectable until many days to weeks later following SARS-CoV-2 infection compared to the synchronous rise in OC43 antibodies. It will be important to expand on this observation with larger numbers of subjects in different age groups to evaluate the extent of SARS-CoV-2 stimulated OC43 memory antibody responses.

Other, preliminary evidence has estimated a negative relationship between HKU1 and OC43 back-boosted responses and the generation of SARS-CoV-2 neutralizing antibodies (47), implying that in some subjects OC43 immune imprinting may have similar effects akin to antigenic epitope masking and/or *original antigenic sin* (48), however, these results were not significant. Whether HCoV immune imprinting has any association with clinical outcomes or SARS-CoV-2 antibody longevity requires further investigation. To our knowledge there is no evidence that HCoV-induced antibodies promote clinical protection in SARS-CoV-2 infected individuals. The vast majority (~98%) of the participants in this study were seropositive for HCoV-OC43; however, they were also seeking treatment at military hospitals for mild to moderate/severe COVID-19. Larger, prospective, longitudinal observational studies in which serum samples are obtained before infection may ultimately be required to definitively determine if HCoV-induced antibodies confer any protection against COVID-19 and the presence of HCoV memory affects the longevity and development of a protective SARS-CoV-2 humoral response.

## Materials And Methods

### Recombinant protein antigens and microsphere coupling

Prefusion stabilized SARS-CoV-2 S-2P glycoprotein ectodomain trimers (hereafter referred to as spike protein) and SARS-CoV-2 RBD were purchased from LakePharma, Inc. (Hopkinton, MA USA). This SARS-CoV-2 spike protein shares an equivalent ectodomain with the NIH Vaccine Research Center designed SARS-CoV-2 S-2P protein, and the Mount Sinai SARS-CoV-2 S-2P protein used in ELISA-based serology (31, 32, 49–51).

Design and expression of prefusion stabilized HCoV-HKU1, HCoV-OC43, SARS-1 and MERS-CoV spike proteins have been previously described (18, 49). A mock antigen, consisting of cell culture supernatant from untransfected HEK cells was collected via centrifugation then filtered through a 0.22 μM PES filter to remove debris. Mock antigen-coupled beads are included in each microtiter well to control for non-specific/artificial antisera binding; samples that react with the mock antigen above an established 3-fold cutoff are retested. Spike proteins were coupled to carboxylated magnetic MagPlex microspheres (Bio-Rad, Hercules, CA) at a protein to microsphere ratio of 15 μg : 100 μL, and antigen-coupled microspheres were resuspended in a final volume of 650 μL following manufacturer’s protocol (Bio-Rad) for amine coupling.

### Non-human primate sera

Archived sera were used from rhesus macaques inoculated with a total dose of 2.6×10^6^ TCID50 of SARS-CoV-2 via a combination of intranasal, intratracheal, oral and ocular inoculation routes (19). Serum samples were collected at dpi 0 (baseline), 1, 3, 5, 7, 10, 12, 14, 17 and 21. To purify serum IgG antibody, 250 μL of serum from each of four experimentally infected NHPs collected 21 dpi were pooled then subjected to thermal inactivation for 30 minutes at 60°C. During inactivation, 2 mL of Protein G agarose, 50% suspension (Sigma-Aldrich, St. Louis, MO, USA) was added to a chromatography column and the bugger was allowed to through. The bead bed was then washed three times with 10 mL of PBS. Inactivated pooled sera were diluted 1:5 in PBS then added to the column. Flow-through was collected, then re-added to the column; this process was repeated for a total of three passes through the column. The bead bed was again washed three times with 10 mL of PBS. Finally, IgG was eluted from the Protein G agarose using a 0.1M Glycine elution buffer, pH 2.5, then returned to neutral pH using 1M Tris-HCl, pH 8.0. Eluted IgG was concentrated using an Amicon Ultra Centrifugal unit (Merck Millipore, Burlington, MA, USA), and the buffer was exchanged to a 1X PBS buffer containing 25% glycerol.

SARS-CoV-2 IgG and IgM antibody seroconversion was determined as the first dpi where a 4-fold increase in the median fluorescence intensity (MFI) was measured compared to the baseline sera collection. Between the 1:250 and 1:1000 dilutions, some NHP IgG antibody reactivities were no longer saturating the upper level of the MMIA. We chose further sera screening at a 1:400 dilution, retaining the ability of the MMIA to detect positives at near MMIA saturation, i.e. >20,000 MFI, while still within the linear region of detection.

### Participant enrollment and sera collection

SARS-CoV-2 negative human serum specimens utilized were from sera collected between 2012 – 2018 in the Infectious Disease Clinical Research Program (IDCRP) Acute Respiratory Infection Consortium Natural History Study (ARIC, IDCRP-045) (24). ARIC sera predate the COVID-19 pandemic and were collected from subjects who had nasopharyngeal swabs tested by nucleic acid amplification methods for virus etiologies of acute respiratory infections; samples collected from individuals with rhinovirus and the seasonal human coronaviruses HCoV-OC43, -HKU1, −229E and -NL63 were used (52). In addition, serum samples were collected since the emergence of SARS-CoV-2 under the IDCRP Epidemiology, Immunology, and Clinical Characteristics of Emerging Infectious Diseases with Pandemic Potential (EPICC, IDCRP-085) protocol; a prospective, longitudinal study to analyze COVID-19 disease. Subjects were enrolled at five hospitals across the continental U.S., including Walter Reed National Military Medical Center (WRNMMC, Bethesda, MD), Brooke Army Medical Center (BAMC, San Antonio, TX), Naval Medical Center San Diego (NMCSD, San Diego, CA), Madigan Army Medical Center (MAMC, Tacoma, WA) and Fort Belvoir Community Hospital (FBCH, Fort Belvoir, VA). Subjects of all race and gender seeking treatment for acute illness at these military hospitals were offered enrollment into the ARIC, IDCRP-045 and EPICC, IDCRP-085 protocols. Study enrollment included subjects with laboratory-confirmed SARS-CoV-2 infection by nucleic acid amplification test, subjects with compatible illness in whom SARS-CoV-2 infection is initially suspected but PCR confirmed as SARS-CoV-2 negative, and asymptomatic subjects at risk of SARS-CoV-2 due to high risk exposure. In this study, 422 serum samples from 204 individual subjects were tested. The earliest serum samples collected ≥ 10 days post-symptom onset (dpso) from subjects with longitudinal samples were included in positive performance agreement analysis. Additionally, serum samples from 35 subjects undergoing treatment at the COVID-19 field hospital at the Jacob K. Javits Convention Center (New York, NY) under the COVID-19 Antibody Prevalence in Military Personnel Deployed to New York (CAMP-NYC) protocol were included in the assessment of assay performance. EPICC, IDCRP-085 and CAMP-NYC protocols were approved by the Uniformed Services University Institutional Review Board.

### Multiplex microsphere-based immunoassay screening procedures

Serum samples were collected from venipuncture in serum separator tubes, processed and stored at −80 °C in 250 μL aliquots until use. For each 96-well plate, a multiplex master mix of antigen-coupled microspheres was made by diluting 100 μL of each antigen-coupled microsphere working stock into 10 mL (1:100) 1XPBS without calcium and magnesium (Corning Inc., Corning, NY) (all mentions of PBS refer to solutions without calcium and magnesium), and 100 μL of this master mix were added to each well so that each well contained 1 μL (~23 ng) of each antigen-coupled microsphere per well. Wells were washed with 1XPBS + 0.05% Tween20 + 0.02% sodium azide two times. One hundred microliters of each serum sample was added to each well. Serum samples were initially diluted within a class II type A2 biological safety cabinet (BSC) then subjected to thermal inactivation for 30 min at 60 °C, further serum dilutions are noted in each respective figure legend. Human serum samples (1.25 μL) were diluted 1:400 in PBS and tested in technical duplicate A and B plates. Controls on each duplicate plate included a PBS blank (wells: A1, B1, G12, H12) and positive (C1, F12) and negative (D1, E12) non-human primate serum. As testing progressed, PCR and serology confirmed human positive/negative samples replaced non-human primate serum samples as the qualified controls for inter- and intra-plate variation.

Samples were incubated at room temperature for 45 minutes with agitation (900 rpm), and plates were washed three times. Secondary antibody (goat anti-human IgG cross-absorbed biotin-conjugated or goat anti-human IgM cross-absorbed biotin-conjugated; Thermo Fisher Scientific, Waltham, MA) was diluted 1:5000 in 1XPBS + 0.05% Tween20 (PBST) and 100 μL of each secondary was added to each well and incubated for 45 minutes with agitation, and plates were washed three times. Streptavidin-phycoerythrin (Bio-Rad) was diluted 1:1000 in PBST and 100 μL was then added to each well and incubated for 30 minutes with agitation, and plates were washed three times. Lastly, 100 μL of PBST was added to each well and plates were resuspended by agitation for 5 minutes. Plates were read on Bio-Plex 200 multiplexing systems (Bio-Rad) with PMT voltage setting to the High RP1 target and 100 bead count requirements. The MFI for the four PBS blank wells on each plate were subtracted from the MFI of each sample well and MFI values for samples are reported as the PBS adjusted average from duplicate plates.

### Threshold cutoffs for SARS-CoV-2 antibody

To establish threshold cutoffs for SARS-CoV-2 spike protein-specific antibody reactivity, we tested 127 archival acute and convalescent human serum samples from ARIC. Acute and convalescent serum samples were collected within approximately three and twenty-eight days of symptom onset, respectively. A cut-off of three times the mean MFI obtained using a mock antigen preparation coupled microsphere was initially used to determine positivity of cross-reactive antibodies in archival serum samples. As cross-reactive antibodies were found to occur in 4 out of 45 serum samples from archival HCoV PCR-positive individuals, we then established a cut-off of three standard deviations above the mean (99.7% probability) MFI of these archival HCoV convalescent serum samples (n= 43) to establish a positivity threshold for detection of SARS-CoV-2 spike protein reactive IgG and IgM antibodies. The remaining 84 archival serum samples were tested against this MFI threshold cutoff for SARS-CoV-2 reactivity. The 127 archival ARIC serum samples were tested in technical duplicates in three independent experiments to establish threshold cutoffs and specificity for SARS-CoV-2.

### Enzyme-linked immunosorbent assay

Flat bottom 96-well microtiter plates (Corning) were coated with 300 ng of SARS-CoV-2 spike protein per well diluted in 100 μl of ELISA coating buffer (1XPBS, 5.3g Na_2_CO_3_, 4.2g NaHCO_3_, pH 9.6) and incubated overnight at 4 °C. The next day, spike protein was removed and 125 μl of 5% BSA blocking buffer were added to each well and incubated for 1 hour at 37 °C. SARS-CoV-2 spike protein coated and blocked plates were then washed three times with 200 μl PBST. Serum samples were subjected to thermal inactivation after being initially diluted in a BSC. Inactivated serum samples were then serially diluted 2-fold in PBS. One hundred microliters of each dilution was added in duplicate to the antigen coated plate, sealed, and incubated at 37 °C for 1 hour. Plates were then washed three times with 200 μl PBST. One hundred microliters of secondary antibody, anti-human (H&L) HRP conjugated, diluted 1:5000 in PBS to each well was added to each well and plates were incubated at 37 °C for 1 hour. Plates were then washed three times with 200 μl PBST. Eighty-five microliters ABST Substrate Solution (Thermo Fisher Scientific) was added to each well and plates were agitated (900 rpm) at room temperature for 30 minutes, then analyzed at 650 nm absorbance on a plate reader (Molecular Devices, San Jose, CA).

### Statistical analysis

Figures were generated and statistical analyses were performed in GraphPad Prism version 7.0. The positive predictive value and negative predictive value were calculated with MedCalc statistical software. ROC analysis was conducted using R version 4.0.2.

## Supplementary Material

Supplement

Supplement

## Figures and Tables

**Table 1.** MMIA SARS-CoV-2 spike protein performance

	SARS-CoV-2 PCR Status/Archival Sera			
SARS-CoV-2 MMIA IgG Antibody Test^2^		Positive^1^	Negative	Total
	Positive	152	0	152
	Negative	3	114	117
	Total	155	114	269
	Sensitivity	98.1%		
	Specificity	100%		

SARS-CoV-2 MMIA IgM Antibody Test^3^	Positive	82	0	82
	Negative	23	114	137
	Total	105	114	219
	Sensitivity 78.1%			
	Specificity 100%			

1 84 archival serum samples and 30 serum samples from PCR negative study enrollees are included as SARS-CoV-2 negative.

2 IgG antibody test included serum samples from n= 155 PCR positive subjects including 82 military treatment facility (MTF) outpatients, 38 MTF hospitalized subjects and 35 Javits Center hospitalized subjects

3 IgM antibody test included serum samples from n= 105 PCR positive subjects including 41 MTF outpatients, 29 MTF hospitalized subjects and 35 Javits Center hospitalized subjects

## References

[1] Petersen E. , Comparing SARS-CoV-2 with SARS-CoV and influenza pandemics. Lancet Infect Dis 20, e238–e244 (2020). 32628905 10.1016/S1473-3099(20)30484-9 PMC7333991

[2] Wu A. , Genome Composition and Divergence of the Novel Coronavirus (2019-nCoV) Originating in China. Cell Host Microbe 27, 325–328 (2020). 32035028 10.1016/j.chom.2020.02.001 PMC7154514

[3] Ibarrondo F. J. , Rapid Decay of Anti-SARS-CoV-2 Antibodies in Persons with Mild Covid-19. N Engl J Med 10.1056/NEJMc2025179 (2020). PMC7397184 32706954

[4] Long Q. X. , Clinical and immunological assessment of asymptomatic SARS-CoV-2 infections. Nat Med 26, 1200–1204 (2020). 32555424 10.1038/s41591-020-0965-6

[5] Seow J. , Longitudinal evaluation and decline of antibody responses in SARS-CoV-2 infection. medRxiv 10.1101/2020.07.09.20148429,2020.2007.2009.20148429 (2020).

[6] Iyer A. S. , Persistence and decay of human antibody responses to the receptor binding domain of SARS-CoV-2 spike protein in COVID-19 patients. Science Immunology 5, eabe0367 (2020). 33033172 10.1126/sciimmunol.abe0367 PMC7857394

[7] Gudbjartsson D. F. , Humoral Immune Response to SARS-CoV-2 in Iceland. N Engl J Med 10.1056/NEJMoa2026116,NEJMoa2026116 (2020). PMC7494247 32871063

[8] Ripperger T. J. , Orthogonal SARS-CoV-2 Serological Assays Enable Surveillance of Low Prevalence Communities and Reveal Durable Humoral Immunity. Immunity 10.1016/j.immuni.2020.10.004 (2020). PMC7554472 33129373

[9] Hartenian E. , The molecular virology of Coronaviruses. J Biol Chem 10.1074/jbc.REV120.013930 (2020). PMC7489918 32661197

[10] U.S. F.D.A (2020, September 11) EUA authorized serology test performance. https://www.fda.gov/medical-devices/coronavirus-disease-2019-covid-19-emergency-useauthorizations-medical-devices/eua-authorized-serology-test-performance

[11] Ayouba A. , Development of a Sensitive and Specific Serological Assay Based on Luminex Technology for Detection of Antibodies to Zaire Ebola Virus. J Clin Microbiol 55, 165–176 (2017). 27795350 10.1128/JCM.01979-16 PMC5228227

[12] Powell R. L. , A Multiplex Microsphere-Based Immunoassay Increases the Sensitivity of SIV-Specific Antibody Detection in Serum Samples and Mucosal Specimens Collected from Rhesus Macaques Infected with SIVmac239. Biores Open Access 2, 171–178 (2013). 23741627 10.1089/biores.2013.0009 PMC3666263

[13] Satterly N. G., Voorhees M. A., Ames A. D., Schoepp R. J., Comparison of MagPix Assays and Enzyme-Linked Immunosorbent Assay for Detection of Hemorrhagic Fever Viruses. J Clin Microbiol 55, 68–78 (2017). 27795340 10.1128/JCM.01693-16 PMC5228264

[14] Kozak R. , Severity of coronavirus respiratory tract infections in adults admitted to acute care in Toronto, Ontario. J Clin Virol 126, 104338 (2020). 32278299 10.1016/j.jcv.2020.104338 PMC7142695

[15] Nickbakhsh S. , Extensive multiplex PCR diagnostics reveal new insights into the epidemiology of viral respiratory infections. Epidemiol Infect 144, 2064–2076 (2016). 26931455 10.1017/S0950268816000339 PMC7113017

[16] Su S. , Epidemiology, Genetic Recombination, and Pathogenesis of Coronaviruses. Trends Microbiol 24, 490–502 (2016). 27012512 10.1016/j.tim.2016.03.003 PMC7125511

[17] Freeman B. , Validation of a SARS-CoV-2 spike protein ELISA for use in contact investigations and serosurveillance. bioRxiv : the preprint server for biology 10.1101/2020.04.24.057323,2020.2004.2024.057323 (2020).

[18] Hicks J. , Serologic cross-reactivity of SARS-CoV-2 with endemic and seasonal Betacoronaviruses. medRxiv 10.1101/2020.06.22.20137695 (2020). PMC7962425 33725211

[19] Munster V. J. , Respiratory disease in rhesus macaques inoculated with SARS-CoV-2. Nature 10.1038/s41586-020-2324-7 (2020). PMC7486227 32396922

[20] Deng W. , Primary exposure to SARS-CoV-2 protects against reinfection in rhesus macaques. Science 369, 818–823 (2020). 32616673 10.1126/science.abc5343 PMC7402625

[21] Lu S. , Comparison of nonhuman primates identified the suitable model for COVID-19. Signal Transduct Target Ther 5, 157 (2020). 32814760 10.1038/s41392-020-00269-6 PMC7434851

[22] Shan C. , Infection with novel coronavirus (SARS-CoV-2) causes pneumonia in Rhesus macaques. Cell Res 30, 670–677 (2020). 32636454 10.1038/s41422-020-0364-z PMC7364749

[23] Kelvin A. A., Zambon M., Influenza imprinting in childhood and the influence on vaccine response later in life. Euro Surveill 24 (2019). 10.2807/1560-7917.ES.2019.24.48.1900720 PMC6891942 31796156

[24] Coles C., Millar E. V., Burgess T., Ottolini M. G., The Acute Respiratory Infection Consortium: A Multi-Site, Multi-Disciplinary Clinical Research Network in the Department of Defense. Mil Med 184, 44–50 (2019). 31778194 10.1093/milmed/usz174 PMC6886571

[25] Norman M. , Ultrasensitive high-resolution profiling of early seroconversion in patients with COVID-19. Nature Biomedical Engineering 10.1038/s41551-020-00611-x (2020). PMC7498988 32948854

[26] Liu L. , A preliminary study on serological assay for severe acute respiratory syndrome coronavirus 2 (SARS-CoV-2) in 238 admitted hospital patients. Microbes and infection 22, 206–211 (2020). 32425648 10.1016/j.micinf.2020.05.008 PMC7233230

[27] Lou B. , Serology characteristics of SARS-CoV-2 infection after exposure and post-symptom onset. European Respiratory Journal 56, 2000763 (2020). 32430429 10.1183/13993003.00763-2020 PMC7401320

[28] Guo L. , Profiling Early Humoral Response to Diagnose Novel Coronavirus Disease (COVID-19). Clinical Infectious Diseases 71, 778–785 (2020). 32198501 10.1093/cid/ciaa310 PMC7184472

[29] Okba N. M. A. , Severe Acute Respiratory Syndrome Coronavirus 2–Specific Antibody Responses in Coronavirus Disease Patients. Emerging Infectious Disease journal 26, 1478 (2020). 10.3201/eid2607.200841 PMC7323511 32267220

[30] Premkumar L. , The receptor-binding domain of the viral spike protein is an immunodominant and highly specific target of antibodies in SARS-CoV-2 patients. Science Immunology 5, eabc8413 (2020). 32527802 10.1126/sciimmunol.abc8413 PMC7292505

[31] Amanat F. , A serological assay to detect SARS-CoV-2 seroconversion in humans. Nat Med 26, 1033–1036 (2020). 32398876 10.1038/s41591-020-0913-5 PMC8183627

[32] Klumpp-Thomas C. , Standardization of enzyme-linked immunosorbent assays for serosurveys of the SARS-CoV-2 pandemic using clinical and at-home blood sampling. medRxiv 10.1101/2020.05.21.20109280 (2020).

[33] Wu F. , Neutralizing antibody responses to SARS-CoV-2 in a COVID-19 recovered patient cohort and their implications. medRxiv 10.1101/2020.03.30.20047365,2020.2003.2030.20047365 (2020).

[34] Tan C. W. , A SARS-CoV-2 surrogate virus neutralization test based on antibody-mediated blockage of ACE2-spike protein-protein interaction. Nat Biotechnol 38, 1073–1078 (2020). 32704169 10.1038/s41587-020-0631-z

[35] Liu L. , Potent neutralizing antibodies against multiple epitopes on SARS-CoV-2 spike. Nature 584, 450–456 (2020). 32698192 10.1038/s41586-020-2571-7

[36] Barnes C. O. , Structures of Human Antibodies Bound to SARS-CoV-2 Spike Reveal Common Epitopes and Recurrent Features of Antibodies. Cell 182, 828–842.e816 (2020). 32645326 10.1016/j.cell.2020.06.025 PMC7311918

[37] Chi X. , A neutralizing human antibody binds to the N-terminal domain of the Spike protein of SARS-CoV-2. Science 369, 650–655 (2020). 32571838 10.1126/science.abc6952 PMC7319273

[38] Robbiani D. F. , Convergent antibody responses to SARS-CoV-2 in convalescent individuals. Nature 584, 437–442 (2020). 32555388 10.1038/s41586-020-2456-9 PMC7442695

[39] Brouwer P. J. M. , Potent neutralizing antibodies from COVID-19 patients define multiple targets of vulnerability. Science 369, 643–650 (2020). 32540902 10.1126/science.abc5902 PMC7299281

[40] Pinto D. , Cross-neutralization of SARS-CoV-2 by a human monoclonal SARS-CoV antibody. Nature 583, 290–295 (2020). 32422645 10.1038/s41586-020-2349-y

[41] Wang L. , Importance of Neutralizing Monoclonal Antibodies Targeting Multiple Antigenic Sites on the Middle East Respiratory Syndrome Coronavirus Spike Glycoprotein To Avoid Neutralization Escape. Journal of Virology 92, e02002–02017 (2018). 29514901 10.1128/JVI.02002-17 PMC5923077

[42] Mayr L. M., Su B., Moog C., Non-Neutralizing Antibodies Directed against HIV and Their Functions. Frontiers in immunology 8, 1590–1590 (2017). 29209323 10.3389/fimmu.2017.01590 PMC5701973

[43] Saphire E. O., Schendel S. L., Gunn B. M., Milligan J. C., Alter G., Antibody-mediated protection against Ebola virus. Nat Immunol 19, 1169–1178 (2018). 30333617 10.1038/s41590-018-0233-9 PMC6814399

[44] Zost S. J. , Potently neutralizing and protective human antibodies against SARS-CoV-2. Nature 584, 443–449 (2020). 32668443 10.1038/s41586-020-2548-6 PMC7584396

[45] Zang J. , Immunization with the receptor-binding domain of SARS-CoV-2 elicits antibodies cross-neutralizing SARS-CoV-2 and SARS-CoV without antibody-dependent enhancement. Cell Discov 6, 61–61 (2020). 32901211 10.1038/s41421-020-00199-1 PMC7471522

[46] Long Q.-X. , Antibody responses to SARS-CoV-2 in patients with COVID-19. Nature Medicine 26, 845–848 (2020). 10.1038/s41591-020-0897-1 32350462

[47] Aydillo T. , Antibody Immunological Imprinting on COVID-19 Patients. medRxiv 10.1101/2020.10.14.20212662,2020.2010.2014.20212662 (2020).

[48] Zarnitsyna V. I. , Masking of antigenic epitopes by antibodies shapes the humoral immune response to influenza. Philos Trans R Soc Lond B Biol Sci 370, 20140248 (2015). 26194761 10.1098/rstb.2014.0248 PMC4528424

[49] Esposito D. , Optimizing high-yifield production of SARS-CoV-2 soluble spike trimers for serology assays. bioRxiv 10.1101/2020.05.27.120204 (2020). PMC7271859 32504802

[50] Kirchdoerfer R. N. , Stabilized coronavirus spikes are resistant to conformational changes induced by receptor recognition or proteolysis. Sci Rep 8, 15701 (2018). 30356097 10.1038/s41598-018-34171-7 PMC6200764

[51] Wrapp D. , Cryo-EM structure of the 2019-nCoV spike in the prefusion conformation. Science 367, 1260–1263 (2020). 32075877 10.1126/science.abb2507 PMC7164637

[52] Bouvier M. , Species-specific clinical characteristics of human coronavirus infection among otherwise healthy adolescents and adults. Influenza Other Respir Viruses 12, 299–303 (2018). 29350887 10.1111/irv.12538 PMC5820427

