# Supplementary material for: A betacoronavirus multiplex microsphere immunoassay detects early SARS-CoV-2 seroconversion and antibody cross reactions
Source: Res Sq. 2020 Nov 24:rs.3.rs-105768. Preprint. [Version 1] doi: 10.21203/rs.3.rs-105768/v1 (PMC7709164; doi:10.21203/rs.3.rs-105768/v1)
Supplement: Supplement [file 6beee00de789505c0500e7c7.pdf]

SUPPLEMENTARY APPENDIX.

A betacoronavirus multiplex microsphere immunoassay detects early SARS-CoV-2 seroconversion and antibody cross reactions

Eric D. Laing[1]*, Spencer L. Sterling[1,3], Stephanie A. Richard[2,3], Nusrat J. Epsi[2,3], Shreshta Phogat[1,3], Emily C. Samuels[1,3], Lianying Yan[1,3], Nicole Moreno[2,3], Christian Coles[2,3], Matthew Drew[4], Jennifer Mehalko[4], Caroline English[2,3], Scott Merritt[2,3,5], Katrin Mende[2,3,5], Kevin K. Chung[6], G. Travis Clifton[5], Vincent J. Munster[7], Emmie de Wit[7], David Tribble[2], Brian K. Agan[2,3], Dominic Esposito[4], Charlotte Lanteri[2], Edward Mitre[1], Timothy H. Burgess[2], and Christopher C. Broder[1]*

Table S1. MMIA SARS-CoV-2 RBD performance

		SARS-CoV-2 PCR Status/Archival Sera		
		Positive	Negative	Total
SARS-CoV-2 MMIA IgG Antibody Test	Positive	135	0	135
	Negative	20	114	134
	Total	155	114	263
	Sensitivity	87.1%		
	Specificity		100%	

Table S2. IgG and IgM seropositivity within 28 days post-symptom onset (dpso)

dspo	IgG+	IgG+/IgM+
7 – 14	80.0% (12/15)	73.3% (11/15)
15 – 28	100% (31/31)	93.5% (29/31)

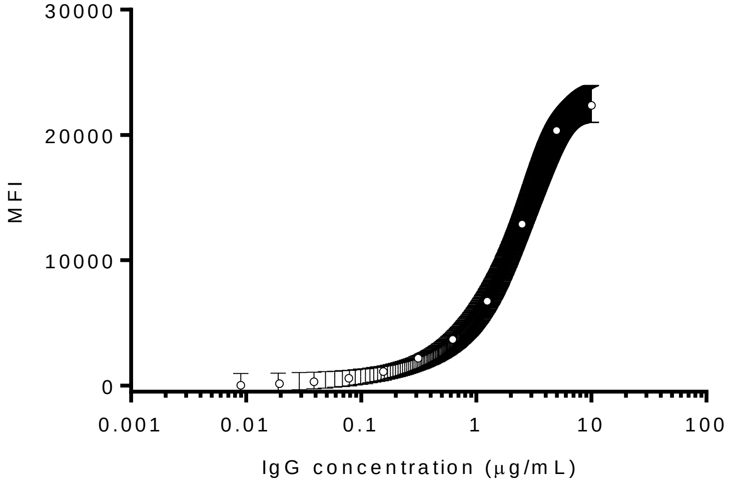

Figure S1. SARS-CoV-2 IgG detection in experimentally challenged NHP serum samples. A sigmoidal curve was used to fit the MEAN±SEM of two independent experiments performed in technical triplicates. MFI, median fluorescence intensities.

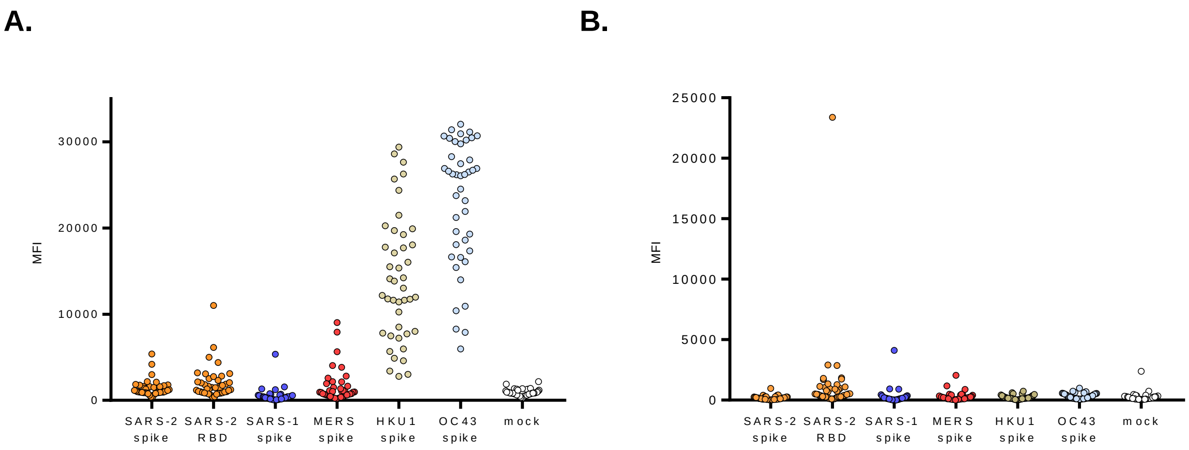

Figure S1. Establishment of 99.7% probability threshold MFI cutoffs for SARS-CoV-2 positive antibodies. Convalescent serum samples (n= 43) from HCoV PCR-positive subjects were tested in the β-CoV MMIA to determine **(A)** IgG antibody and **(B)** IgM antibody reactivity to SARS-CoV-2 spike protein and RBD with a pre-2019 sera bank. Data represent the MEAN of three independent experiments performed in technical duplicates.

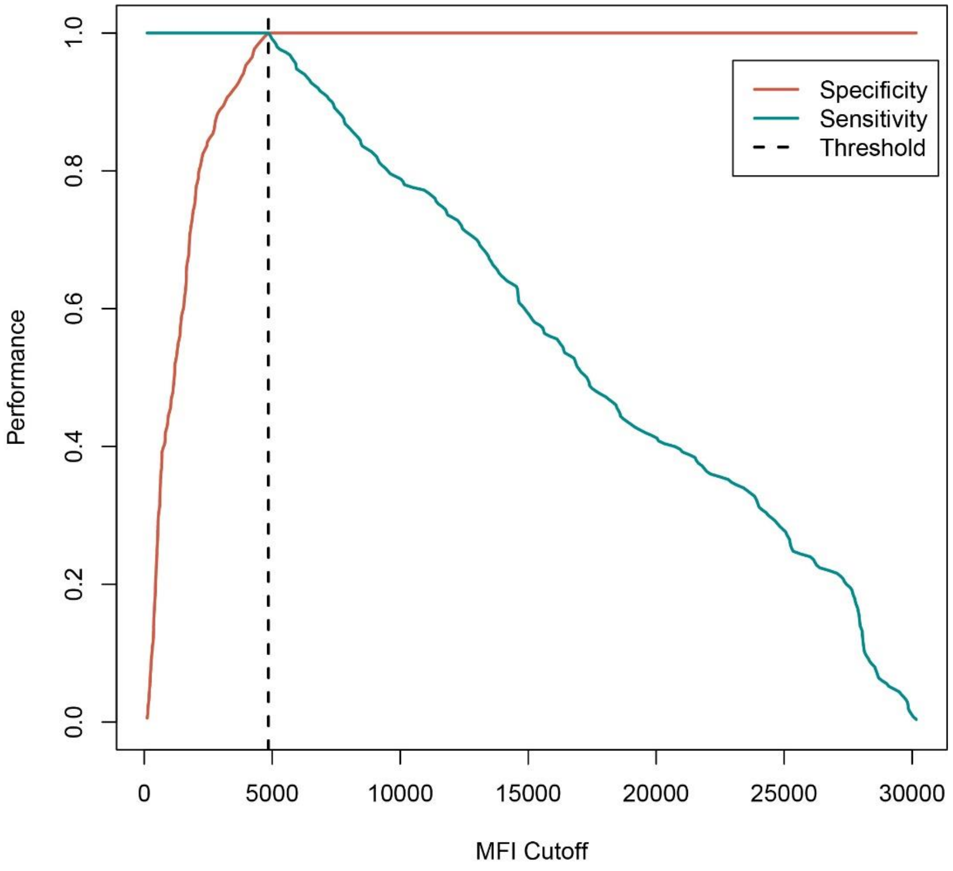

Figure S3. Receiver operating characteristic curve analysis of SARS-CoV-2 spike protein IgG antibody reactivity in a β-CoV MMIA. PCR-confirmed SARS-CoV-2 positive and negative serum samples (n= 422) were tested with β-CoV MMIA and 100% was achieved at threshold cutoff of 4854 MFI.

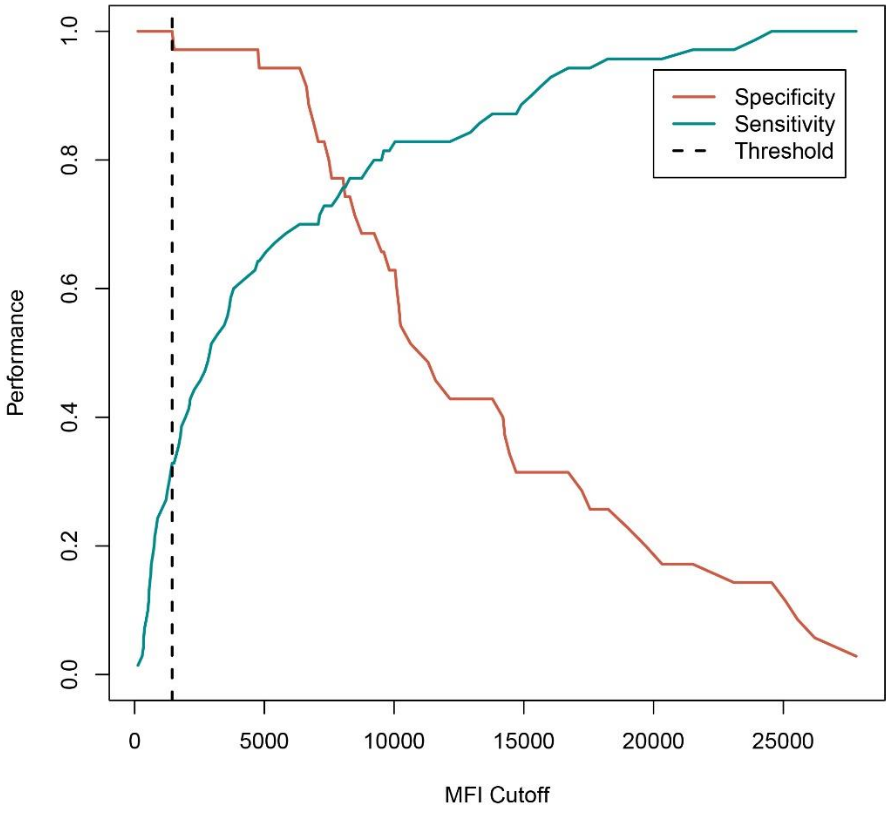

Figure S4. Receiver operating characteristic curve analysis of SARS-CoV-2 spike protein IgM antibody reactivity in a β-CoV MMIA. PCR-confirmed SARS-CoV-2 positive and negative serum samples (n= 105) were tested with β-CoV MMIA and 100% specificity was achieved at threshold cutoff of 1446 MFI.

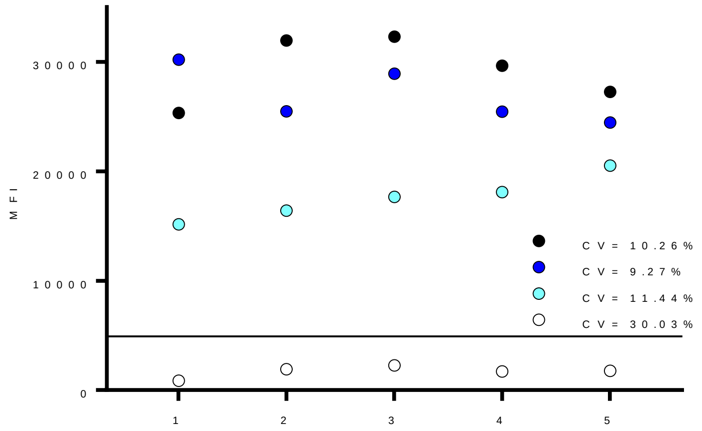

Figure S5. Positive and negative results are reproducible over independent MMIA tests. Selected positive(s) and negative serum samples were tested across independent experiments. CV, coefficient of variation, percentages are indicated on the graphs for each sample. A solid line indicates the threshold cutoff for positive IgG.
